# Is there a role for retinoids to treat minimal residual disease in neuroblastoma?

**DOI:** 10.1054/bjoc.2000.1430

**Published:** 2000-11

**Authors:** K K Matthay, C P Reynolds

**Affiliations:** Department of Pediatrics, University of California San Francisco School of Medicine, San Francisco, CA, 94143; Department of Pediatrics, University of Southern California School of Medicine and Children’s Hospital Los Angeles, Los Angeles, CA, 90027, USA

## Abstract

A variety of pre-clinical and clinical data point toward high drug levels of retinoids being required to achieve optimal efficacy against neuroblastoma. The results of the Kohler trial reported in this issue demonstrate that low-dose 13-cis-RA does not have clinical efficacy against neuroblastoma in a setting of minimal residual disease. A comparison of the Kohler trial with the US CCG trial provides clinical evidence that high-dose levels of retinoids are optimal for treating minimal residual disease in neuroblastoma. The comparison of high-dose and low-dose 13-cis-RA studies in neuroblastoma suggests the intriguing possibility that high dose, pulse schedules of other retinoids could be effective as therapeutic and chemopreventive agents in diseases where low-dose, chronic retinoid administration was not effective. Pre-clinical and perhaps clinical studies of the latter concept should be considered. © 2000 Cancer Research Campaign


*British Journal of Cancer* (2000) **83**(9), 1121–1123
© 2000 Cancer Research Campaign
doi: 10.1054/ bjoc.2000.1430, available online at http://www.idealibrary.com on IDEAL®

Editorial

### Is there a role for retinoids to treat minimal residual disease in neuroblastoma?

**KK Matthay and CP Reynolds**

Department of Pediatrics, University of California San Francisco School of Medicine, San Francisco, CA 94143 and Department of Pediatrics, University of Southern California School of Medicine and Children's Hospital Los Angeles, Los Angeles, CA 90027, USA

**Summary** A variety of pre-clinical and clinical data point toward high drug levels of retinoids being required to achieve optimal efficacy against neuroblastoma. The results of the Kohler trial reported in this issue demonstrate that low-dose 13-cis-RA does not have clinical efficacy against neuroblastoma in a setting of minimal residual disease. A comparison of the Kohler trial with the US CCG trial provides clinical evidence that high-dose levels of retinoids are optimal for treating minimal residual disease in neuroblastoma. The comparison of high-dose and low-dose 13-cis-RA studies in neuroblastoma suggests the intriguing possibility that high dose, pulse schedules of other retinoids could be effective as therapeutic and chemopreventive agents in diseases where low-dose, chronic retinoid administration was not effective. Pre-clinical and perhaps clinical studies of the latter concept should be considered. © 2000 Cancer Research Campaign

### Rationale for retinoids in neuroblastoma

Relapse from minimal residual disease occurs in over 50% of patients with high-risk neuroblastoma, despite intensive multi-modality therapy with haematopoietic stem cell support (Matthay et al, 1993). To improve outcome, therapies are required with novel mechanisms effective against residual tumour that was able to survive myeloablative doses of cytotoxic agents. The retinoid 13-cis-retinoic acid (13-cis-RA), also known as isotretinoin, is an isomer of all-trans-retinoic acid (ATRA) that occurs naturally, but in very low concentrations, and has been employed for therapy and chemoprevention of cancer (Smith et al, 1992). In vitro, both 13-cis-RA and ATRA caused differentiation, decreased proliferation and decreased *MYCN* expression in neuroblastoma cell lines, including some established from tumours refractory to cytotoxic chemotherapy (Sidell, 1982; Thiele et al, 1985; Sidell et al, 1986; Reynolds et al, 1991, 1994; Abemayor, 1992; Melino et al, 1997). Because of the strong activity of ATRA in acute promyelocytic leukaemia, an in vitro comparison was performed of the clinically achievable levels of 13-cis-RA (5 μM) and ATRA (0.5 μM). It was shown in 6 of 12 neuroblastoma cell lines that for ATRA and 13-cis-RA at clinically achievable levels, the two drugs were equal in activity, while for 6 of 12 lines 13-cis-RA had significantly better activity than did ATRA (Reynolds et al, 1994).

Anecdotal reports as well as a U.S. Children's Cancer Group (CCG) phase II trial of 13-cis-RA in children with neuroblastoma suggested only modest activity in recurrent disease when 13-cis-RA was given continuously at 100 mg/m$^2$/day (Reynolds et al, 1991; Finklestein et al, 1992). In vitro testing of 13-cis-RA using multiple neuroblastoma cell lines demonstrated that levels of 5–10 μM caused growth arrest, which was sustained in some cell lines for weeks after removal of the 13-cis-RA from the culture medium. These data suggested that high-dose, pulse 13-cis-RA would be effective for clinical studies in neuroblastoma patients and might allow dose escalation above levels obtainable with continuous dosing. Based on the results of the in vitro modelling, a phase I dose escalation trial was designed in which patients were given higher doses of 13-cis-RA on an intermittent schedule, to allow recovery from toxicity (Villablanca et al, 1995). This phase I study was done in children with high-risk neuroblastoma following autologous bone marrow transplantation, and established that the high-dose intermittent schedule (using 13-cis-RA for 14 days consecutively out of every 28 days) had low toxicity and achieved levels known to be effective against neuroblastoma in vitro. The maximum tolerated dose was 160 mg/m$^2$ daily, which achieved peak levels of 7 μM. Three complete responses in bone marrow were observed in ten evaluable patients (Villablanca et al, 1995). These data showed that 13-cis-RA is well tolerated after intensive chemoradiotherapy, and suggested that it could have efficacy against minimal residual disease that causes relapse.

### Phase III clinical trials of 13-cis-RA in neuroblastoma

Based on the laboratory and clinical studies of 13-cis-RA, the U.S. CCG designed a Phase III randomized trial for high-risk neuroblastoma. Children who were progression-free after completion of either intensive chemotherapy or myeloablative chemoradiotherapy and autologous bone marrow transplantation were assigned to either 6 months of high-dose intermittent 13-cis-RA or to no further treatment. The event-free survival (EFS) for the group randomized to 13-cis-RA ($n$ = 130) was 46%, significantly higher than that of patients randomized to no further treatment ($n$ = 128), at 29% ($P$ = 0.027) (Matthay et al, 1999).

In this issue of *British Journal of Cancer*, Kohler and colleagues for the European Neuroblastoma Study Group (ENSG) report a double-blind randomized trial of low dose continuous 13-cis-RA given after completion of cytotoxic therapy for high-risk neuroblastoma. The results of the ENSG study differ from that of the CCG trial in that no difference in EFS was seen with the use of

*Correspondence to*: KK Matthay

13-cis-RA given to patients in complete or very good partial remission after cytotoxic therapy. There are a number of possible reasons for this discrepancy. The most likely reason for the lack of efficacy in the ENSG trial is the low dose employed for 13-cis-RA. The study was begun in 1989, prior to publication of the data from the in vitro studies and the phase I trial that led to the CCG randomized study. The ENSG study was designed using a dose that was approximately 15% of that shown to be the maximum tolerated dose in the phase I study by Villablanca and colleagues (Villablanca et al, 1995) and of the subsequent randomized CCG phase III trial (Matthay et al, 1999). At that low dose, drug levels would be far below those shown to be effective for sustained growth arrest of neuroblastoma cell lines (Reynolds et al, 1991, 1994; Reynolds and Lie, 2000).

Other differences, which may have influenced the outcome, include the somewhat later start of the 13-cis-RA in the European trial, at a median of 341 days from diagnosis, compared to an average of 290 days in the Children's Cancer Group study. Beginning 13-cis-RA relatively soon after cytotoxic therapy, before tumour cells can begin to grow, may be critical for this agent to be most effective. The greater efficacy of 13-cis-RA against minimal disease (compared to a larger tumour burden) is supported by the fact that the CCG study showed that the most significant effect of the 13-cis-retinoic acid was in children in apparent complete remission, with no significant difference seen when the analysis was restricted to those in partial remission (Matthay et al, 1999). Although the ENSG study selectively treated only children whose disease was in complete or very good partial remission after chemotherapy, it is possible that the longer interval from ablative chemotherapy to the use of the differentiating agent allowed regrowth of tumour. The new US cooperative paediatric clinical trials group, the Children's Oncology Group, will test 13-cis-RA after myeloablative therapy even earlier, beginning 8 weeks after haematopoietic stem cell transplantation.

Compliance with the use of 13-cis-RA is a potential problem in small children, since currently the only available formulation is in capsules. For small children, the dosing may be sub-optimal, with the inability to take the capsules requiring removal from the capsule and administration with food, as done in the CCG study, or else not administering to very young children, as in the ENSG study. Since no pharmacokinetic measurements were obtained in the Kohler study, compliance cannot be verified. In the Kohler study, both induction regimens and myeloablative therapy (employed in 126 of the 175 randomized patients) were treatment centre-dependent, leading to a considerable variation in the different therapies given to patients prior to beginning 13-cis-RA. Furthermore, patient numbers were smaller in the ENSG study relative to the US CCG study. All of these issues could have diminished the power of the ENSG study to identify a positive benefit for 13-cis-RA.

The pre-clinical and clinical data on use of 13-cis-RA against neuroblastoma indicate that dosing, scheduling, and beginning treatment with 13-cis-RA at a time when tumour burden is low are all important elements in the efficacy of 13-cis-RA. The failure of 13-cis-RA to improve survival in the Kohler study provides further evidence supporting the use of optimal doses and schedules of retinoids when employed as anti-neoplastic agents.

### NEW RETINOIDS AND POSSIBLE FUTURE RETINOID COMBINATIONS

Although 13-cis-RA improved the survival of patients with high-risk neuroblastoma, resistance to 13-cis-RA and ATRA occurs in neuroblastoma. If agents can be identified that are effective against retinoic acid-resistant neuroblastoma at drug levels obtainable in patients, further improvements in survival may be achieved (Reynolds and Lie, 2000). N-(4-hydroxyphenyl)-retinamide (4-HPR), or fenretinide, is a synthetic retinoid that is cytotoxic for tumour cells. In contrast to 13-cis-RA and ATRA, 4-HPR does not induce maturational changes, but causes apoptosis (Delia et al, 1993; Ziv et al, 1994; Supino et al, 1996) and 4-HPR has shown activity against cell lines known to be resistant to ATRA (Delia et al, 1993; Sheikh et al, 1995; Kazmi et al, 1996; Supino et al, 1996). 4-HPR has been reported to inhibit the growth of neuroblastoma cell lines in vitro at 4-HPR concentrations of 1–10 μM in a dose dependent manner (Di Vinci et al, 1994; Mariotti et al, 1994; Ponzoni et al, 1995), and 4-HPR was highly active against RA-resistant neuroblastoma cell lines at 5 to 10 μM drug levels (Reynolds et al, 1997).

Although until recently 4-HPR was only used at low doses, toxicity of 4-HPR in clinical trials has been minimal (Cobleigh et al, 1993; Formelli et al, 1993; Costa et al, 1995) and no haematologic toxicity has been reported. The major clinical toxicity of 4-HPR is decreased night vision, due to decreased plasma retinol levels (Decensi et al, 1997). Initial results of our US CCG phase I trial in children have shown no systemic toxicity of oral 4-HPR to date, even at higher doses that have achieved 4-HPR plasma levels of 3 to 7.5 μM (Basniewski et al, 1999). A phase II trial of oral 4-HPR as a single agent in recurrent neuroblastoma is planned within the Childrens Oncology Group, once the ongoing CCG phase I trial is complete.

Recent studies have shown that 5–10 μM 4-HPR stimulates large increases of ceramide in neuroblastoma cell lines, which is likely one of the mechanisms by which anti-tumour cytotoxicity is achieved with 4-HPR (Maurer et al, 1999). Neuroblastoma cell lines established at relapse after myeloablative therapy often have acquired a sustained resistance during the course of therapy to drugs which act via traditional cytotoxic mechanisms (Keshelava et al, 1998). However, such cell lines can be sensitive to high levels of 4-HPR, perhaps due to 4-HPR achieving tumour cell cytotoxicity via novel mechanisms of action (Maurer et al, 1999). The activity of 4-HPR against drug-resistant neuroblastoma cell lines, including cell lines resistant to ATRA and 13-cis-RA (Reynolds et al, 1997), suggests that high-dose 4-HPR may be effective against tumour cells that persist after current therapeutic approaches. Future trials may also employ 4-HPR in combination with agents that modulate ceramide metabolism so as to increase the anti-tumour activity of 4-HPR (Maurer et al, 2000).

### REFERENCES

Abemayor E (1992) The effects of retinoic acid on the in vitro and in vivo growth of neuroblastoma cells. *Laryngoscope* **102**: 1133–1149

Basniewski PG, Reid JM, Villablanca JG, Reynolds CP and Ames MM (1999) A phase I pharmacokinetic study of fenretinide (HPR) in chldren with high-risk solid tumors. A Children's Cancer Group (CCG) study. *Proc Am Assoc Cancer Res* **40**: 92

Cobleigh MA, Dowlatshahi K, Deutsch TA, Mehta RG, Moon RC, Minn F, Benson ABd, Rademaker AW, Ashenhurst JB, Wade, JLd et al. (1993) Phase I/II trial of tamoxifen with or without fenretinide, an analog of vitamin A, in women with metastatic breast cancer. *J Clin Oncol* **11**: 474–477

Costa A, De Palo G, Decensi A, Formelli F, Chiesa F, Nava M, Camerini T, Marubini E and Veronesi U (1995) Retinoids in cancer chemoprevention. Clinical trials with the synthetic analogue fenretinide. *Ann NY Acad Sci* **768**: 148–162

 © 2000 Cancer Research Campaign

Decensi A, Fontana V, Fioretto M, Rondanina G, Torrisi R, Orengo MA and Costa A (1997) Long-term effects of fenretinide on retinal function. *Eur J Cancer* **33**: 80–84

Delia D, Aiello A, Lombardi L, Pelicci PG, Grignani F, Grignani F, Formelli F, Menard S, Costa A, Veronesi U et al. (1993) N-(4-hydroxyphenyl)retinamide induces apoptosis of malignant hemopoietic cell lines including those unresponsive to retinoic acid. *Cancer Res* **53**: 6036–6041

Di Vinci A, Geido E, Infusini E and Giaretti W (1994) Neuroblastoma cell apoptosis induced by the synthetic retinoid N-(4-hydroxyphenyl)retinamide. *Int J Cancer* **59**: 422–426

Finklestein JZ, Krailo MD, Lenarsky C, Ladisch S, Blair GK, Reynolds CP, Sitarz AL and Hammond GD (1992) 13-cis-retinoic acid (NSC 122758) in the treatment of children with metastatic neuroblastoma unresponsive to conventional chemotherapy: report from the Childrens Cancer Study Group. *Med Pediatr Oncol* **20**: 307–311

Formelli F, Clerici M, Campa T, Di Mauro MG, Magni A, Mascotti G, Moglia D, De Palo G, Costa A and Veronesi U (1993) Five-year administration of fenretinide: pharmacokinetics and effects on plasma retinol concentrations. *J Clin Oncol* **11**: 2036–2042

Kazmi SM, Plante RK, Visconti V and Lau CY (1996) Comparison of N-(4-hydroxyphenyl)retinamide and all-trans-retinoic acid in the regulation of retinoid receptor-mediated gene expression in human breast cancer cell lines. *Cancer Res* **56**: 1056–1062

Keshelava N, Seeger RC, Groshen S and Reynolds CP (1998) Drug resistance patterns in human neuroblastoma cell lines derived from patients at different phases of therapy. *Cancer Res* **58**: 5396–5405

Mariotti A, Marcora E, Bunone G, Costa A, Veronesi U, Pierotti MA and Della Valle G (1994) N-(4-hydroxyphenyl)retinamide: a potent inducer of apoptosis in human neuroblastoma cells. *J Natl Cancer Inst* **86**: 1245–1247

Matthay KK, Atkinson JB, Stram DO, Selch M, Reynolds CP and Seeger RC (1993) Patterns of relapse after autologous purged bone marrow transplantation for neuroblastoma: a Childrens Cancer Group pilot study. *J Clin Oncol* **11**: 2226–2233

Matthay KK, Villablanca JG, Seeger RC, Stram DO, Harris RE, Ramsay NK, Swift P, Shimada H, Black CT, Brodeur GM, Gerbing RB and Reynolds CP (1999) Treatment of high-risk neuroblastoma with intensive chemotherapy, radiotherapy, autologous bone marrow transplantation, and 13-cis-retinoic acid. Children's Cancer Group. *N Engl J Med* **341**: 1165–1173

Maurer BJ, Metelitsa LS, Seeger RC, Cabot MC and Reynolds CP (1999) Increase of ceramide and induction of mixed apoptosis/necrosis by N-(4-hydroxyphenyl)- retinamide in neuroblastoma cell lines [see comments]. *J Natl Cancer Inst* **91**: 1138–1146

Maurer BJ, Cabot MC and Reynolds CP (2000) Modulators of ceramide metabolism syngergize fenretinide cytotoxicity in multiple tumor cell types. *Proc Amer Assoc Cancer Res* **41**: 239

Melino G, Thiele CJ, Knight RA and Piacentini M (1997) Retinoids and the control of growth/death decisions in human neuroblastoma cell lines. *J Neurooncol* **31**: 65–83

Ponzoni M, Bocca P, Chiesa V, Decensi A, Pistoia V, Raffaghello L, Rozzo C and Montaldo PG (1995) Differential effects of N-(4-hydroxyphenyl)retinamide and retinoic acid on neuroblastoma cells: apoptosis versus differentiation. *Cancer Res* **55**: 853–861

Reynolds CP and Lie S (2000) Retinoid therapy of neuroblastoma. In *Neuroblastoma*, Brodeur GM, ST, Tsuchida Y and Voute PA (ed) pp. 519–540. Elseviere Science: Amsterdam

Reynolds CP, Kane DJ, Einhorn PA, Matthay KK, Crouse VL, Wilbur JR, Shurin SB and Seeger RC (1991) Response of neuroblastoma to retinoic acid in vitro and in vivo. *Prog Clin Biol Res* **366**: 203–211

Reynolds CP, Schindler PF, Jones DM, Gentile JL, Proffitt RT and Einhorn PA (1994) Comparison of 13-cis-retinoic acid to trans-retinoic acid using human neuroblastoma cell lines. *Prog Clin Biol Res* **385**: 237–244

Reynolds CP, Melton LJ and Wang W (1997) N-(4-hydroxyphenyl-retinamide is highly active against retinoic acid resistant neuroblastoma cell lines. *Proc Am Assoc Cancer Res* **38**: 25

Sheikh MS, Shao ZM, Li XS, Ordonez JV, Conley BA, Wu S, Dawson MI, Han QX, Chao WR and Quick T (1995) N-(4-hydroxyphenyl)retinamide (4-HPR)-mediated biological actions involve retinoid receptor-independent pathways in human breast carcinoma. *Carcinogenesis* **16**: 2477–2486

Sidell N (1982) Retinoic acid induced growth inhibition and morphologic differentiation of human neuroblastoma cells in vitro. *J Natl Cancer Inst* **68**: 589–593

Sidell N, Sarafian T, Kelly M, Tsuchida T and Haussler M (1986) Retinoic acid-induced differentiation of human neuroblastoma: a cell variant system showing two distinct responses. *Exp Cell Biol* **54**: 287–300

Smith MA, Parkinson DR, Cheson BD and Friedman MA (1992) Retinoids in cancer therapy. *J Clin Oncol* **10**: 839–864

Supino R, Crosti M, Clerici M, Warlters A, Cleris L, Zunino F and Formelli F (1996) Induction of apoptosis by fenretinide (4HPR) in human ovarian carcinoma cells and its association with retinoic acid receptor expression. *Int J Cancer* **65**: 491–497

Thiele CJ, Reynolds CP and Israel MA (1985) Decreased expression of N-myc precedes retinoic acid-induced morphological differentiation of human neuroblastoma. *Nature* **313**: 404–406

Villablanca JG, Khan AA, Avramis VI, Seeger RC, Matthay KK, Ramsay NK and Reynolds CP (1995) Phase I trial of 13-cis-retinoic acid in children with neuroblastoma following bone marrow transplantation. *J Clin Oncol* **13**: 894–901

Ziv Y, Gupta MK, Milsom JW, Vladisavljevic A, Brand M and Fazio VW (1994) The effect of tamoxifen and fenretinide on human colorectal cancer cell lines in vitro. *Anticancer Res* **14**: 2005–2009

*© 2000 Cancer Research Campaign*

